# Attitudes and preferences in patients with acromegaly on long-term treatment with somatostatin analogues

**DOI:** 10.1530/EC-16-0038

**Published:** 2016-07-25

**Authors:** Cecilia Follin, Sven Karlsson

**Affiliations:** Department of EndocrinologySkåne University Hospital, Lund, Sweden

**Keywords:** attitudes, acromegaly, long-term treatment, somatostatin analogues

## Abstract

**Introduction:**

Patients with acromegaly can be treated with surgery, medical therapy and/or radiation therapy. For the patients not being cured with surgery, treatment with somatostatin analogues (SSAs) is the primary therapy. SSA can be taken by self- or partner-administered injections in addition to being given by a nurse at a clinic. The aim was to assess if patients with acromegaly prefer self-injections and to investigate their attitudes towards long-term medical therapy.

**Method:**

All patients in the southern medical region of Sweden with a diagnosis of acromegaly and treated with SSA were eligible for the study (*n* = 24). The study is based on a questionnaire asking about the patients’ attitudes and preferences for injections with SSA, including their attitudes towards self-injection with SSA.

**Results:**

The patients’ (23 included) median age was 68.5 years and the patients had been treated with SSA for 13 (1–38) years. One patient was currently self-injecting. All of the other patients were receiving injections from a nurse at a clinic. Three patients preferred self-injections, one preferred partner injections and 19 patients did not prefer self- or partner injections. The most frequent arguments to not preferring self-injections were ‘feeling more secure with an educated nurse’ and ‘preferring regular contact with a specialised nurse’.

**Conclusion:**

Patients with acromegaly prefer regular contact with the endocrine team to the independence offered by self-injections. These findings might mirror the patients’ desires for continuity and safety. We need to address patients’ concerns regarding injections with SSA and support them in their choices.

## Introduction

Acromegaly is a rare chronic condition caused by excess growth hormone (GH) secretion – usually from a pituitary tumour – and it has an incidence rate of only 3.3 cases per million people. The mean age at diagnosis is 40 years with an equal number of men and women being affected (1). Uncontrolled disease is associated with an approximately 72% increase in mortality compared with the general population (2) and with multiple comorbidities such as osteoarthritis, type 2 diabetes and hypertension (3, 4). The aims of treatment for acromegaly are to control/reduce tumour size, normalise GH and insulin-like growth factor 1 (IGF-1) levels and to improve comorbidities. Current treatments consist of surgery, medical therapy and/or radiation therapy. The only therapy that has a potential to cure is surgical resection of the GH-secreting adenoma, but surgery is only successful in about 60% of patients (5). Consequently, the high rate of persistent GH hypersecretion after surgery necessitates chronic medical treatment. Current medical therapies available to treat acromegaly consist of somatostatin analogues (SSAs) and dopamine agonist (DA) medication, both of which act to suppress GH secretion from pituitary adenomas or remnant tissue and thus lead to reduced IGF-1 levels and reduced symptoms (6, 7). A GH-receptor antagonist (e.g. pegvisomant) is also an option, and such drugs block the effects of GH at the level of the GH receptor and also reduce IGF-1 levels and control symptoms. Pegvisomant is used in combination with SSA to gain increased efficacy (8). For the patients who are not cured with surgery, long-term treatment with SSA is the primary therapy used to achieve long-term remission and biochemical control (9).

In patients with acromegaly, impaired quality of life (QoL) – as assessed by specific questionnaires – has been reported even in patients who experience long-term cure (10). The chronic need for monthly injections of SSA might negatively impact QoL due to long-term dependence on medical care and having only a passive role in one’s self-care. SSA can be administered as an intra-muscular injection(s) or as a deep subcutaneous injection(s). Because lanreotide autogel is supplied in prefilled syringes and is injected into deep subcutaneous tissue, it is possible for patients with acromegaly to use self- or partner-administered injection (11), and this offers the possibility to lead a more independent life. Further, injections of lanreotide have been shown to be given reliably and safely outside a health care clinic and are considered as an alternative to injections by health care professionals for motivated patients (12, 13, 14, 15). In the study by Salvatori and coworkers, the primary end-point of which was to evaluate the efficacy and safety of self- or partner administration, 70% of the participants were able to self-inject and 30% to partner inject correctly after training (13). However, unsupervised self-injections do not appeal to all patients, and Bevan and coworkers showed that the patients who are more likely to self-inject are younger patients of working age (14). Our clinical experience is that limited numbers of patients with acromegaly use self- or partner administration of SSA even though this option has been available in Sweden since 2001. Acromegaly has been associated with loss of initiative, depression, low self-esteem and social withdrawal (16), and these conditions might negatively impact the patient’s ability to take a more active part in their treatment and thus gain increased independence. Self- or partner injections might be a way to increase the patient’s independence and reduce the number of clinic visits. The aim of this study was to use questions with multiple choices to assess whether patients with acromegaly on continuous treatment with SSA prefer self-injections or prefer injections administered by health care professionals and to investigate their attitudes towards long-term medical therapy. We hypothesised that patients without comorbidities prefer self- or partner injections compared with patients with comorbidities, as the latter group might have an increased need of health care. Thus, we also aimed to describe the patients’ clinical characteristics during long-term treatment with SSA.

## Subjects and methods

### Design

This study is based on a questionnaire asking about the patients’ attitudes and preferences for injections with SSA, including their attitudes towards self-injection with SSA. This is the first study using the present questionnaire. The questionnaire is developed by a nurse specialised in endocrinology and a senior endocrinologist (C Follin and S Karlsson). The questionnaire was reviewed by one nurse with great clinical experience of acromegaly together with the medical advisor and the team at Ipsen company. The authors made the final decision on the questionnaire without involvement from Ipsen. The questionnaire was first sent to three patients to investigate if they understood the questions and were able to answer accordingly. Based on the three patients’ comments on the questionnaire, the authors made some minor changes, which made it more clear, and the questionnaire was then sent to all participants of the study. The questions can be found in Table 1.

**Table 1:** Structure of the questionnaire in this study.

The participants can mark Yes or No:
1. Are you educated in health care?
2. Where do you currently receive your injections? (type of clinic)
3. Have you ever been informed about the possitbility to self-inject?
4. Have you ever been educated in self-injections?
5. Do you currently self- or partner inject? (with the possibility to separate between self- and partner injection)
6. After education in the technique of self-injections, would you like to self-inject or partner inject?
The participants mark the statements that match their reasons for not being willing to self- or partner inject (they can mark more than one):
It feels unpleasant to self-inject or partner inject.
Afraid of needle-stick and injections.
Feel secure with an educated nurse.
Afraid of inacccurate self-injection.
I don’t want to lose the regular contact with a nurse.
I don’t know.
The participants mark the statements that match their reasons for being willing to self- or partner inject (they can mark more than one):
I am more independent with self-injections.
Do not need to book appointments at the hospital.
Possibility to influence my situation.
Reduce the number of visits to the clinic.
Take an active role in my own care.
I don’t know.
Additional comments (free text)

Previous treatment for acromegaly and current treatment for complications related to acromegaly or its treatment such as pituitary deficiency, type 2 diabetes, hypertension and cardiac failure were extracted from the medical records of the participants. The study was approved by the regional ethical committee in Sweden (DNR 2015/2).

### Participants

This study recruited patients from the four centres in the southern medical region of Sweden (population of 1.3 million people) currently treating patients with acromegaly. All patients (*n* = 24) with a documented diagnosis of acromegaly (defined as an IGF-1 level above the age-related normal range or GH level >0.4 ng/mL after an oral glucose tolerance test) and being treated with SSA and with the ability to communicate in Swedish were eligible for the study. One patient was excluded due to cognitive dysfunction. Thus, of 24 eligible patients, 23 patients (10 men and 13 women) were included in the study.

All patients were sent an information letter, the questionnaire and an informed consent form to sign. The patients who did not return the informed consent and questionnaire (*n* = 5) were phoned by one of the authors asking if they were willing to participate. The remaining five patients then sent in their consent form and completed questionnaire.

### Laboratory measurements

Laboratory data were based on the participants’ most recent visit to the endocrinology clinic at 2–8 months before the questionnaires were sent to the participants. Biochemical control of their disease was based on a single serum sample of IGF-1, and the patients were considered to be controlled if their IGF-1 level fell within the age-related normal range. All four centres used the same laboratory for measuring IGF-1 and it was measured with a chemiluminescent immunoassay. The normal range was 71–239 µg/L in subjects aged 31–42 years and 60–179 µg/L in subjects aged 42–70 years (inter-assay coefficient of variation (CV)%, 8% at the level of 30 µg/L and 8% at the level of 239 µg/L).

### Pituitary function and comorbidities

Pituitary function was recorded in all patients based on current pituitary hormone replacement therapy. Comorbidities were recorded as treatment of hypertension, type 2 diabetes or cardiac failure.

### Data analysis

The results of the questionnaire, medical history and clinical characteristics were analysed using SPSS version 21.0. Data are presented as the frequency or as the median and range (min–max).

## Results

### Demographic and clinical characteristics of the patients

Twenty-three patients with a mean age of 68.5 (34–81) years when completing the questionnaire were included. The patients’ median age at diagnosis of acromegaly was 49.5 (28–72) years, and they were enrolled in this study on average 13.5 (1–38) years after diagnosis. Among these patients, 17/23 had been treated with surgery and three patients had been treated with radiotherapy. All patients were treated with SSA with a median duration of 13 (1–27) years. In addition to SSA, five patients were treated with DA and two were treated with a GH-receptor antagonist. No patients were treated with GH. One patient was undergoing hormone replacement with testosterone, thyroxine and cortisone; one had replacement with testosterone and cortisone; three had replacement with thyroxine; and two had replacement with cortisone (Table 2).

**Table 2:** Characteristics including previous treatment and hormone substitution in 23 patients with acromegaly.

	**Patients** (*n* = 23)
	**Median** (range)
Men/women (*n*)	10/13
Current age (years)	68.5 (34–81)
Age at diagnosis (years)	49.5 (28–72)
Years since diagnosis	13.5 (1–38)
Surgery (no of patients)	16
Surgery (quantity/patient)	1 (0–5)
Radiotherapy (*n*)	3
Somatostatin therapy (*n*)	22
Duration of somatostatin therapy (years)	13 (1–27)
Dopaminagonist (*n*)	5
GH-receptor antagonist (*n*)	2
GH therapy (*n*)	0
Testosterone substitution (*n*)	4
Thyroxine substitution (*n*)	4
Cortisone substitution (*n*)	3
Hypertension (*n*)	15
Diabetes type 2 (*n*)	3
Cardiac failure	1

GH, growth hormone; *n*, numbers.

### Biochemical control in patients

Twenty-one patients were biochemically controlled as indicated by serum IGF-1 levels within the reference range, and two patients presented with serum IGF-1 levels above the reference range.

### Frequency of comorbidities

15/23 (65%) of the patients were being treated for hypertension, and among these patients, three were also being treated for type 2 diabetes and one was being treated for heart failure. An additional two patients without hypertension were being treated for type 2 diabetes (Table 2).

### Patients’ attitudes towards self-administration

Of the 23 patients, one was self-administrating the SSA and none were using their partners for injections. Fifteen patients were receiving SSA injections by a nurse in an endocrinology clinic, and seven were receiving injections in an outpatient clinic. When asked about preferences of self- or partner administration, three patients said they like to self-inject (including the patient who was using self-injection at the time) and one likes to use partner administration. Two of the patients were educated in health care (a physician and a nurse) and they had been treated for 10 and 20 years with SSA. The third patient who stated that she was willing to self-inject had been on SSA for 10 years and was in working age. The patient who liked to use his partner for injections was 34 years old and diagnosed and treated with surgery 1 year before the study.

**Figure 1:**
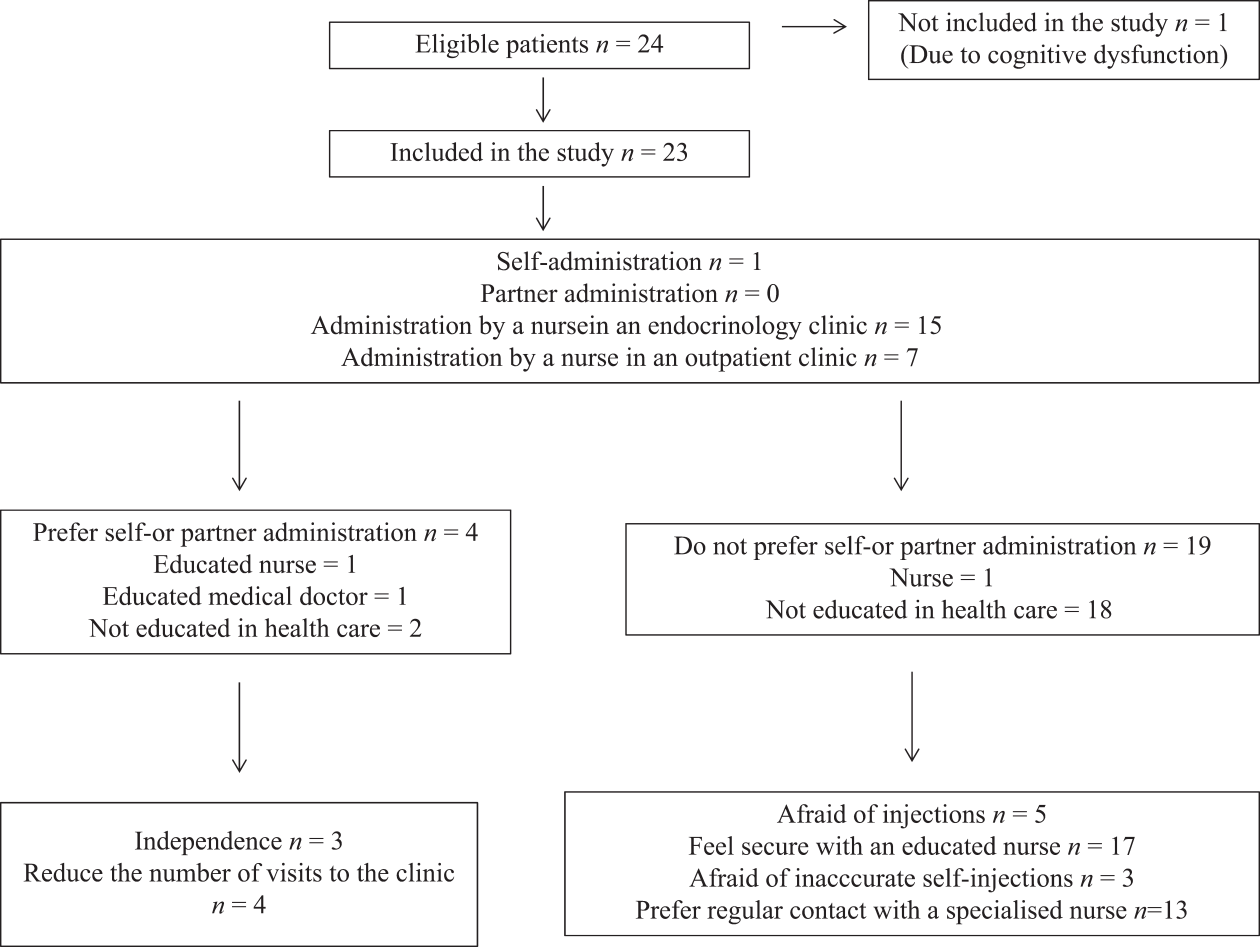
Flowchart of included patients and their attitudes towards self-administration.

Nineteen of twenty-three patients did not want to use self- or partner administration. The most frequent arguments to not preferring self-injections were ‘Feel secure with an educated nurse’ and ‘Prefer regular contact with a specialised nurse’. The most frequent reasons for preferring self-injections were ‘I am more independent with self-injections’ and ‘Reduce the number of visits to the clinic’ (Fig. 1).

### Comorbidities and additional treatments in patients who prefer self-administration

The only patient who self-injected was also being treated for diabetes and hypertension. Two of the patients who were willing to self-inject were on treatment for hypertension. The only patient who was willing to use partner injection was being treated with SSA only. None of the patients who preferred self- or partner injection were being treated with DA or GH-receptor antagonists.

## Discussion

In this study, we found that 20/23 (87%) of the patients with acromegaly being treated with SSA prefer that the injection be administered by an educated nurse, and these patients reported that they wanted to feel safe and to have regular contact with a specialised nurse. Lanreotide has raised the possibility of home administration, which might be beneficial to patients who are being treated with SSA. The potential benefits might be to reduce the negative impacts of their disease in their daily life and to reduce the amount of time they have to spend in the clinic. However, we found that the large majority of patients preferred to have the injection administrated by a nurse, and one explanation could be that they would like to maintain continuity, safety and clinical connections. Based on our findings and past reports, we speculate that patient preferences are (impacted) by life situations, expectations and concerns and may not be easily predicted by a simple, easy-to-use formula (17). In other studies, patients with acromegaly report concerns about the medication and with acceptance of the disease and its consequences (18, 19). These patients have also reported unmet needs regarding care, such as insufficient information about the impact of the disease. Furthermore, they tend to report experiencing the injections as an unpleasant experience (18), and having a negative attitude towards medication has been shown to be related to more negative perceptions of one’s illness and worse QoL (20). It is often a struggle for these patients to make their medical treatments fit their life schedules, and this was shown in a previous study reporting that patients with acromegaly sometimes take ‘drug holidays’ to feel free (19). These concerns might impact the patients’ attitudes towards chronic treatment with SSA and, in particular, to self-administration.

Previous studies of self- or partner administration of SSA in patients with acromegaly report that patients are able and willing to self- or partner inject (12, 13, 14, 15), which is in complete contrast to this study and to the authors’ own clinical experience. The differences in patients’ attitudes might be explained by the patients’ younger age and shorter length of treatment with SSA in the previous studies compared with this study (12, 13, 14, 15). It has been reported that treatment adherence and personal understanding of the disease were worse in patients with acromegaly after a longer duration of follow-up compared with patients with a shorter duration of follow-up (19). It should also be pointed out that this study is based on a questionnaire without immediately offering training in self- or partner administration. This is in contrast to previous studies where the investigators directly approached the patients (12, 13, 14). The present results simply reflect the patients’ attitudes in a routine clinical practice rather than a controlled study in which the key end-points would be to evaluate the efficacy and safety of self- or partner administration after training.

It has been shown that patients with other diagnoses than acromegaly like to have the possibility to ask questions about the problems and concerns that affect them and their daily life (20). Consequently, if the visit to the endocrinologist is combined with regular visits with the endocrine nurse, then the entire endocrine team may offer an enhanced feeling of continuity and safety for patients. This might improve the patients’ coping with the chronic treatment and improve their QoL. In addition, the endocrinologists will also be provided with a holistic knowledge of the patients’ condition. If we are able to add the patients’ perspectives, we will be able to offer better health care (21).

This study provides clinical data after a mean duration of 13 years of treatment with SSA. All included patients, except two, had their symptoms under control according to measurements of serum IGF-1 levels. We found a high proportion of patients also receiving treatment for hypertension. Hypertension is considered to be a frequent complication with acromegaly, and it affects approximately one-third of all patients, but with a wide range of 17–57% depending on the study population (22, 23, 24). The high prevalence of hypertension in our patients might be explained by the long-term duration of the disease and the advanced age of the patients.

The strengths and potential limitations of this study merit consideration. This study investigated the patients’ attitudes solely through a questionnaire, which limited the variation in the responses compared with performing interviews with the patients. The questionnaire asked about the patients’ attitudes towards self- or partner injections, and this is the first questionnaire we are aware of for investigating this specific task. The questionnaire was not formally validated, but the validity was strengthened by internal expert review by a nurse and an endocrinologist and also three patients who contribute to its relevance for this population. The strength of this study was its very high response rate in which 23 out of 24 eligible patients accepted to participate.

In conclusion, these data suggest that the majority of the patients with acromegaly prefer regular contact with the endocrine team rather than independence in terms of self-injections. However, the option of self-injections should still be offered to the patients. These findings might mirror the patients’ requirements for continuity and safety and we need to address the patients’ concerns regarding injections with SSA. We need to support them in their daily life when living with a chronic disease by offering regular contact with the endocrine team as part of a supportive care programme that is designed to meet the patients’ specific needs.

## Declaration of interest

The authors declare that there is no conflict of interest that could be perceived as prejudicing the impartiality of the research reported.

## Funding

This study was sponsored by Ipsen Nordic.
